# Investigation of external quality factor and coupling coefficient for a novel SIR based microstrip tri-band bandpass filter

**DOI:** 10.1371/journal.pone.0258386

**Published:** 2021-10-25

**Authors:** Abdul Basit, Muhammad Irfan Khattak, Jamel Nebhen, Atif Jan, Gulzar Ahmad

**Affiliations:** 1 Electrical Engineering Department, University of Engineering and Technology, Peshawar, Pakistan; 2 College of Computer Engineering and Sciences, Prince Sattam bin Abdulaziz University, Alkharj, Saudi Arabia; University of Scranton, UNITED STATES

## Abstract

In this article, a new method is developed to design a three-band miniaturized bandpass filter (BPF) that uses two asymmetrically coupled resonators with one step discontinuity and open-circuited uniform impedance resonator (UIR) to achieve Global Interoperability with Microwave Access (WiMAX) and Radio Frequency Identification (RFID) applications. First, a pair of asymmetrical step impedance resonators (ASIR) is used to implement a dual band filter, then a half wavelength uniform impedance resonator is added below to the transmission line to achieve a triple band response. The proposed filter resonates at frequencies of 3.7 GHz, 6.6 GHz, and 9 GHz with the fractional bandwidth of 7.52%, 5.1%, and 4.44%, respectively. By adjusting the physical length ratio (α) and the impedance ratio (R) of the asymmetric SIR, the proposed fundamental frequencies of the triple BPF are obtained. Moreover, the coupling coefficient (K_e_) and external quality factor (Q_e_) are investigated between the resonators and the input/output ports of the transmission line and are calculated using full-wave EM simulator HFSS. In addition, five transmission zeros are introduced near the passbands to increase the filter selectivity. Finally, the proposed filter is designed and fabricated with a size of 13.69 × 25 mm (0.02 λ_g_ × 0.03 λ_g_), where λ_g_ represents the guiding wavelength in the first passband. The simulated and measured results have a good correspondence, thus confirming the design concept.

## Introduction

The field of microwave and RF communication continuously demands a compact wireless transceiver for commercial products, especially in the combination of IEEE 802.11b/g (GSM), IEEE 802.11a (WLAN), GPS, RFID, 3G, 4G, Bluetooth, and automotive radar system. These wireless standards require high data rates and large bandwidth in the radio frequency spectrum. Besides mobile phones, RF systems are also necessary for scientific instruments, navigation and even in medical applications. Thus, efforts are carried out to design such a compact and low power consumption component which simultaneously allows the wireless standards smoothly without interference with other RF bands. One of the key components in such a system is the compact and high selectivity bandpass filters and its performance dominates the entire microwave communication system. For this, the design of multiband BPFs with compact size and low insertion loss plays an important role in the multiband wireless transceiver and it constitutes a great challenge for the circuit designers [1–7]. In the past several dual and triple bandpass filters were intensively proposed and investigated by combining two or more single BPFs, a stub loaded resonator (SLR), a step impedance resonator (SIR) with one or more step discontinuities, and the multimode resonators (MMR’s). For example, in [8–14] several dual-band filters were designed at different resonance frequencies with their own merits and demerits, however, poor selectivity, larger circuit dimensions, and high insertion losses were the major drawbacks associated with these designs.

The design of filters based on SIR with one step discontinuity allows to better control the spurious bands as compared to the traditional SIR which has two-step discontinuities causes more losses and larger circuit size. It has the advantages of designing higher-order compact BPFs with high selectivity and low insertion losses such as dual, tri, quad or quintuple BPFs because of its inherently higher-order resonant modes [15]. A tri-band BPF loaded with pi-section SIR is presented in [16], for GPS (Link-2), WIMAX, and WLAN applications with their merit of greater bandwidth but filter selectivity, insertion losses, and larger size still need to be improved. In [17], another triple-band response is achieved using asymmetric stub loaded resonators for Wireless Medical Telemetry Service (WMTS), WLAN, and WiMAX applications with a shortcoming of high insertion loss (IL), low fractional bandwidth (FBW), poor selectivity as well as larger circuit dimensions. To improve the isolation between the passbands as well as the passband insertion losses, a high-frequency selectivity triple-band BPF is designed and fabricated on Rogers RO-4003 material in [18], using a novel multimode resonator for WCDMA, WiMAX, and WLAN wireless applications. The filter has its strong merit of greater bandwidth and low IL, but the dimension of the filter still needs to be improved and the circuit complexity also increases using MMR’s. In [19], the authors utilize the double mode to design a filter which gives three passbands for GSM and GPS applications. The filter shows a good response for insertion loss as well as high-frequency selectivity by exciting six TZ’s between the bands, but larger circuit size is a major drawback associated with the design. Another three working bands filter is designed in [20], using composite Right Left-Handed (CRLH) resonator on Roger RO-4003C substrate material with dielectric constant 3.38. The presented filter has a serious issue in FBW, circuit size, poor isolation between the passbands as well as insertion losses especially for the first and third passbands which is greater than -3dB. To overcome the size problem and make the filter suitable for compact wireless transceivers, a high selectivity dual and tri-band filter in [21], is designed and implemented on Rogers substrate material using a common resonator feeding technique. The presented filter has good passband insertion loss however, FBW and circuit size need to be upgraded. In [22], asymmetric T-shaped SLR’s based tri passband filter is designed for 2.48/3.58/4.48 GHz wireless applications with wider FBW and low in-band IL, however, larger circuit area and poor isolation between the passbands was the major drawbacks. The authors of [23, 24], designed a triple band BPF’s using SIR’s structure. Both the filter has good passband selectivity, but larger circuit dimensions were a major drawback associated with the design. Moreover, the later one has poor IL as well as narrow FBW. Recently a UIR based triband filter is designed and implemented in [25]. The proposed design has a larger circuit area, poor FBW and greater insertion losses. To improve the passbands IL, a compact three passband filter is designed using ring MMR’s in [26]. The proposed filter has good selectivity, but larger circuit area is still a challengeable problem for researcher. The authors of [27–29], designed a triband filter using embedded resonators and stub loaded square ring resonators for different wireless applications, but high insertion losses, narrow bandwidth, and larger circuit area are the major drawbacks associated with the designs.

Targeting the IEEE 802.16 (WiMAX) and RFID wireless applications, this paper proposes an ultra-compact tri-band BPF using two asymmetrically coupled resonators with one-step discontinuity and a uniform impedance resonator, centered at 3.7 GHz, 6.6 GHz, and 9 GHz with the fractional bandwidth of 7.52%, 5.1% and 4.44%, respectively. The first and second passbands are made by asymmetrically coupled step impedance resonators (SIR), while the third passband is made by a half-wavelength uniform impedance resonator. The resonant frequency of the BPF is determined by adjusting the physical length ratio (α) and the impedance ratio (R) of the asymmetric SIR. Also, the coupling coefficient is determined by the gap between two resonators and a pair of 50 Ω input/output ports. Finally, the proposed three-band BPF was fabricated on the Rogers substrate and the simulated results are in good agreement with the measured results. This article introduces an easy way to design ultra-compact three passbands filter without complicated design and manufacturing processes.

## Resonance conditions of the proposed resonator

**Fig 1:**
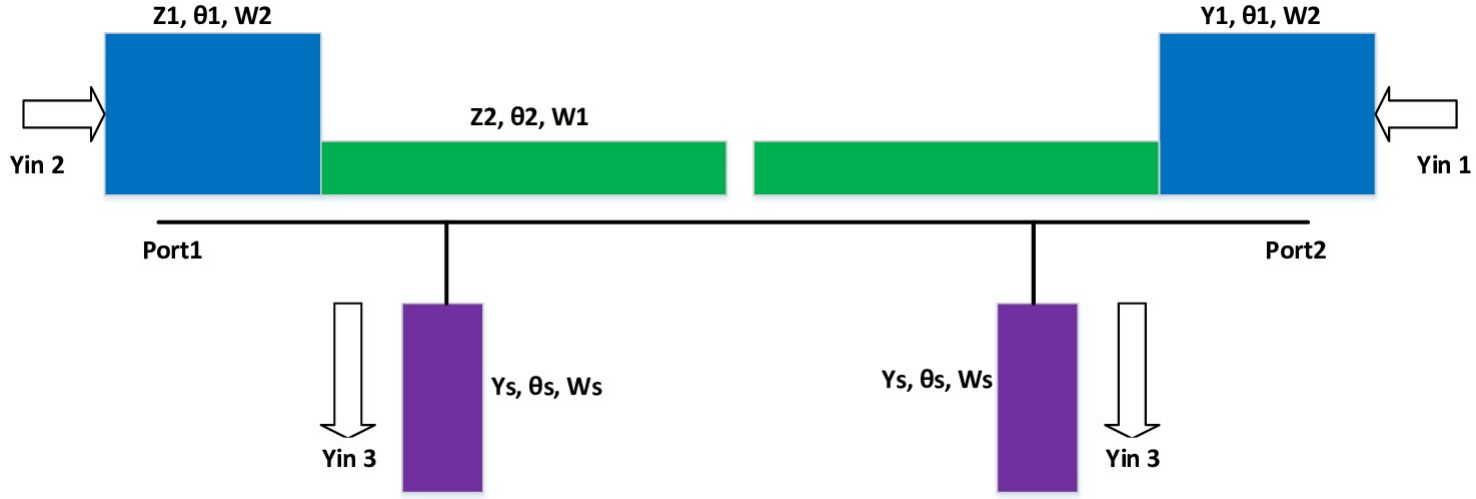
Proposed asymmetric SIR configuration.

**Fig 2:**
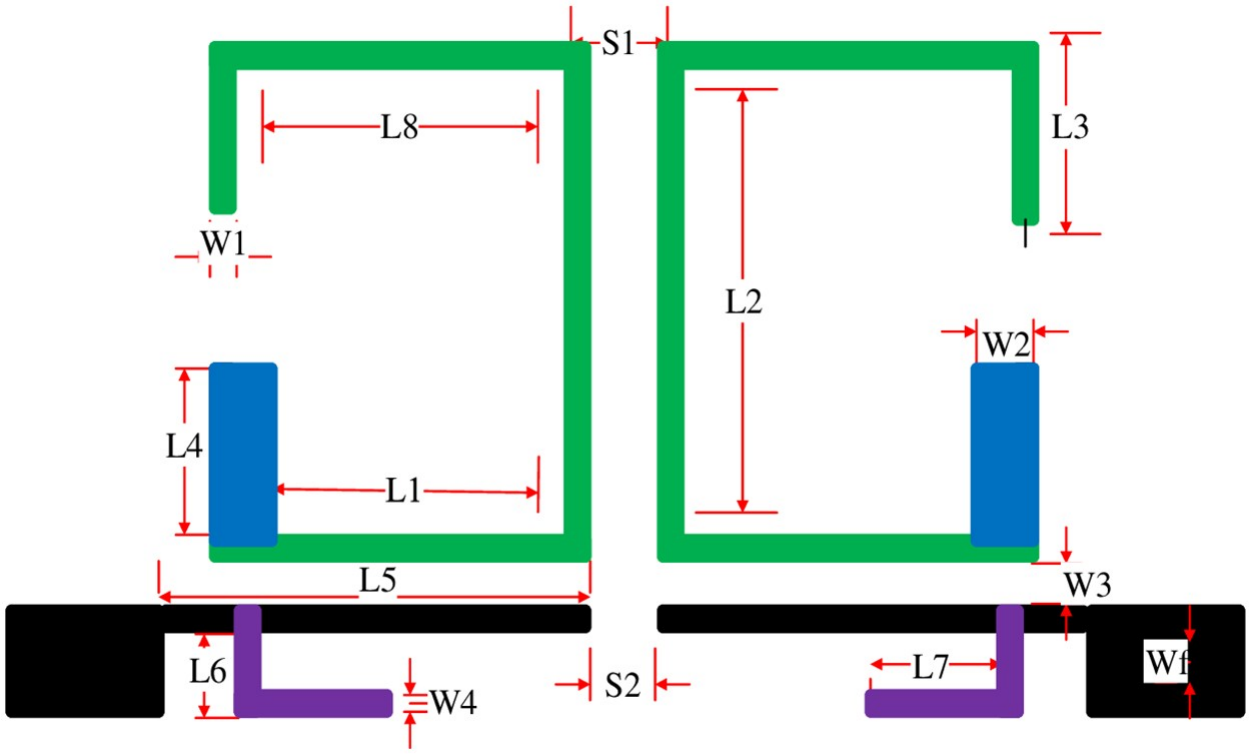
Proposed topology of the tri-band filter.

The basic structure of the asymmetric SIR is illustrated in Fig 1. It consists of a low impedance section (Z_1_) cascaded with a high impedance section (Y_2_) and are bent in a ring-like shape to reduce the circuit size as shown in Fig 2. The two ring-like shape SIRs are responsible for the generation of the first and second passbands, while the resonator having uniform admittance (Y_s_) that is attached to the 50 Ω input/output ports is responsible for the generation of the third passband, respectively. The proposed configuration has a one-step discontinuity as compared to the conventional SIR which has two-step discontinuity, due to this arrangement the harmonics of the fundamental resonance frequencies can be shifted easily far away without increasing the circuit size or increasing the discontinued step impedance sections. The length and width of the low and high impedance section are denoted by L_1_, W_2_, and L_2_, W_1_ with characteristic impedance Z_1_ = 1/Y_1_ and Z_2_ = 1/Y_2_, respectively, whereas θ_1_ and θ_2_ represent the electrical lengths of the high impedance section and low impedance section of the microstrip line as shown in Fig 1. The physical length ratio (α) and the impedance ratio (R) of the asymmetric SIR can be defined as follows [15];

$$\alpha =\frac{\theta_{2}}{\theta_{1}+\theta_{2}}=\frac{\theta_{2}}{\theta_{t}}$$ (1)

Here *θ*_*t*_ is the total wavelength of the asymmetric step impedance resonator.

and

$$R=\frac{Z_{2}}{Z_{1}}=\text{tan}\theta_{1}\text{tan}\theta_{2}$$ (2)

In above equations

$$\theta_{1}=\beta_{1}l_{1}=\left(2\pi /\lambda \right)l_{1}=\left(2\pi /{\text{v}}_{\text{p}}\right)\text{f}l_{1}$$ (3)

and

$$\theta_{2}=\beta_{2}l_{2}=\left(2\pi /\lambda \right)l_{2}=\left(2\pi /{\text{v}}_{\text{p}}\right)\text{f}l_{2}$$ (4)

where f denotes the frequency and v_p_ is the phase velocity of the microstrip line. The characteristic input admittance Y_in_ of the asymmetric SIR seen from the open-end can be found by neglecting the effect of discontinuities and are follows;

$${\text{Y}}_{\text{in}}={\text{Y}}_{\text{in1}}+{\text{Y}}_{\text{in2}}$$ (5)

$$Y_{in}{}_{1}=Y_{3}\frac{jY_{1}\text{tan}\theta_{1}+jY_{3}\text{tan}\theta_{3}}{Y_{2}-Y_{1}\text{tan}\theta_{1}\text{tan}\theta_{3}}$$ (6)

$$Y_{in}{}_{2}=Y_{2}\frac{jY_{1}\text{tan}\theta_{1}+jY_{2}\text{tan}\theta_{2}}{Y_{2}-Y_{1}\text{tan}\theta_{1}\text{tan}\theta_{2}}$$ (7)

and

$${\text{Y}}_{\text{in3}}=\text{j}\;{\text{Y}}_{\text{s}}\;\text{tan}\;\theta_{\text{s}}$$ (8)

The resonance condition occurs when equating below equation to zero i.e.


$$\text{Im}\left({\text{Y}}_{\text{in}}\right)=0$$ (9)

For simplicity suppose Y_2_ = Y_3_ = Y_s_. Substituting (5)–(8) into (9), the resonance condition can be written as;

$$2kC_{1}(1-C_{\alpha T}C_{(1-\alpha )T})+(K^{2}-C_{1}{}^{2})(C_{\alpha T}+C_{(1-\alpha )T})=0$$ (10)

and

$$Cs(k-C_{1}C_{(1-\alpha )T})(k-C_{1}C_{\alpha T})=0$$ (11)

where C_1_ = tanθ_1_, CαT = tanα θ_T_, C(1-α) T = tan(1-α) θ_T_, and Cs = tanθ_s_, respectively. Based on (10) and (11), the proposed filter applications such as WIMAX and RFID are achieved simultaneously by choosing the appropriate impedance ratio (R = 0.45) and physical length ratio (α = 0.71), respectively.

## Triband filter geometry

An ultra-compact tri-passband filter having 13.69×25 mm (0.02 λ_g_×0.03 λ_g_) or 0.0006 λ^2^_g_ circuit size where λ_g_ represents the guided wavelength at first passband, consisting of two asymmetric SIRs and one UIR, fabricated on Rogers RO-4350 substrate having relative permittivity 3.66, thickness 0.762 mm, and tested on Agilent E5071C network analyzer is presented in this study. The proposed triband filter is simulated using full-wave electromagnetic software HFSS-13. The proposed filter topology is shown in Fig 2 while the geometrical circuit parameters are listed in Table 1, respectively. The first and second passband centered at 3.7 GHz and 6.6 GHz are obtained by the two coupled asymmetric SIRs consist of high impedance section (Z_1_) and low impedance section (Z_2_) for WiMAX and RFID applications, while the third passband centered at 9 GHz is achieved through UIR which is attached to the input/output port transmission line.

**Table 1:** Proposed triband filter geometrical parameters in millimeter (mm).

L_1_	6.8	L_2_	11	L_3_	3.7	L_4_	5	L_5_	10
L_6_	1	L_7_	4.5	L_8_	6	W_1_	0.23	W_2_	1.4
W_3_	0.25	W_4_	0.8	S_1_	0.7	S_2_	0.2	W_f_	1.7

## Coupling coefficient and external quality factor

In this study, a compact tri-passband filter is designed and fabricated using two asymmetric SIRs and one UIR. The two asymmetric SIRs are designed in such a manner that it produces the desired coupling coefficient and quality factor with the input/output port of the microstrip line. Thus, the coupling coefficient and quality factor can be determined by the space S_1_ between the two resonators which is fixed to 0.7 mm and the gap W_3_ between the pair of asymmetric SIRs and the input/output ports transmission line. When the gap between the two resonators increases, the external quality factor increases while the coupling coefficient decreases and vice versa. The Q and the K_e_ can be determined by performing the parametric analysis for different values of S_1_ and W_3_ using a 3D EM full-wave simulation. After performing the parametric analysis, the quality factor coefficient (Q) and the coupling coefficient (K_e_) can be found using the following expression [30];

$$Q=\frac{f_{c}}{FBW}$$ (12)

$$K_{e}=\frac{f_{h}^{2}-f_{l}^{2}}{f_{h}^{2}+f_{l}^{2}}$$ (13)

**Fig 3:**
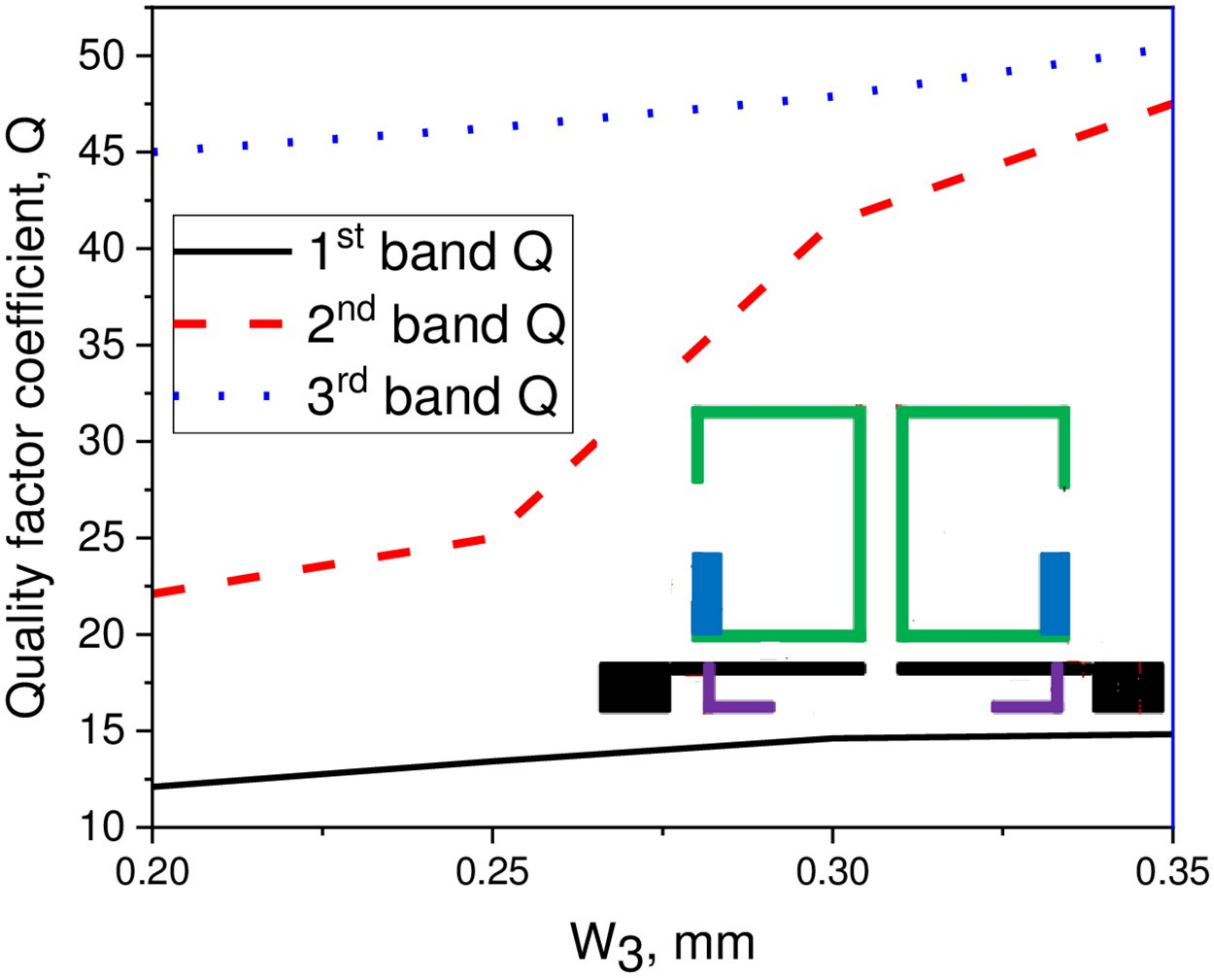
Graph of quality factor coefficient with W_3_ gap.

**Fig 4:**
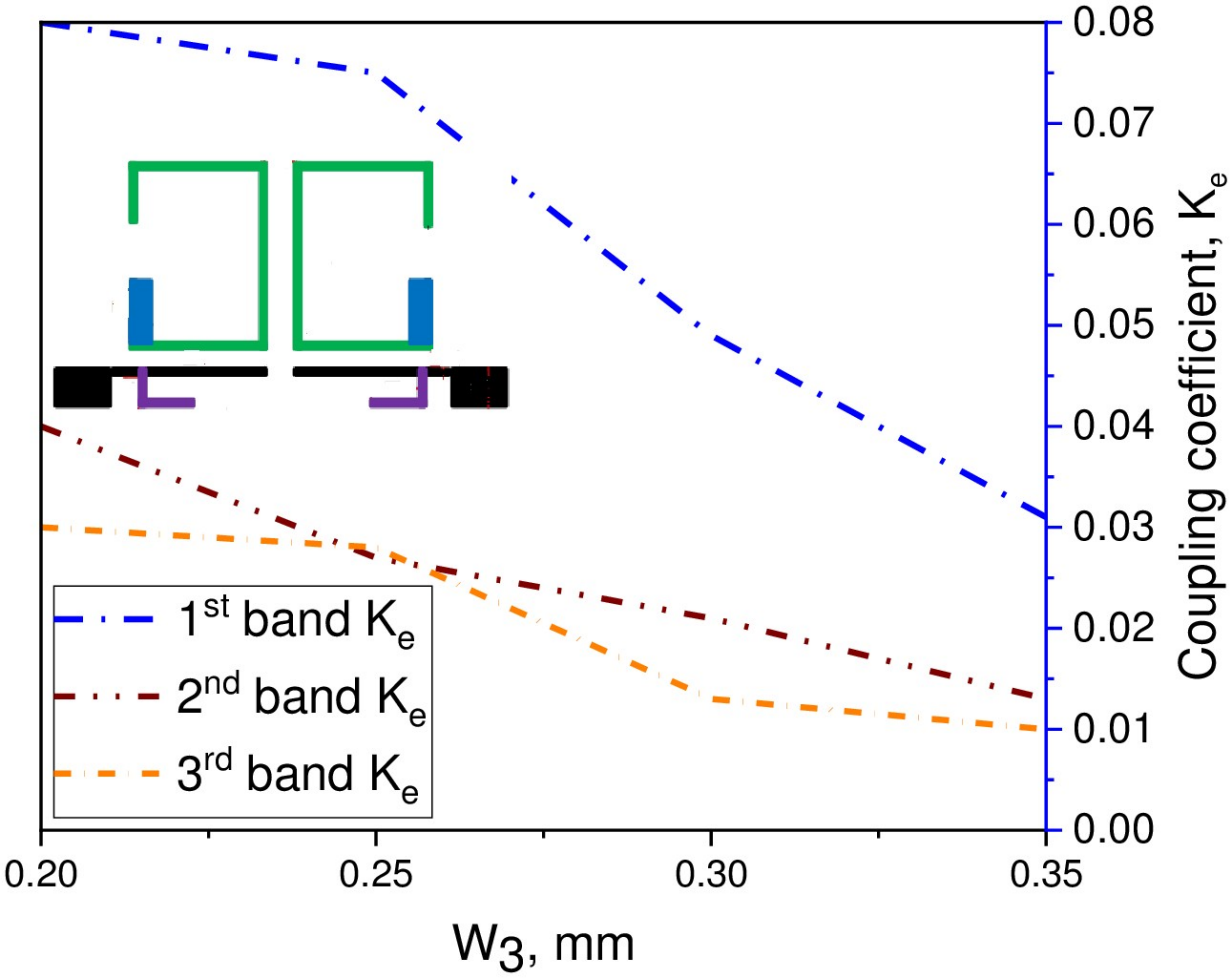
Graph of coupling coefficient with W_3_ gap.

**Fig 5:**
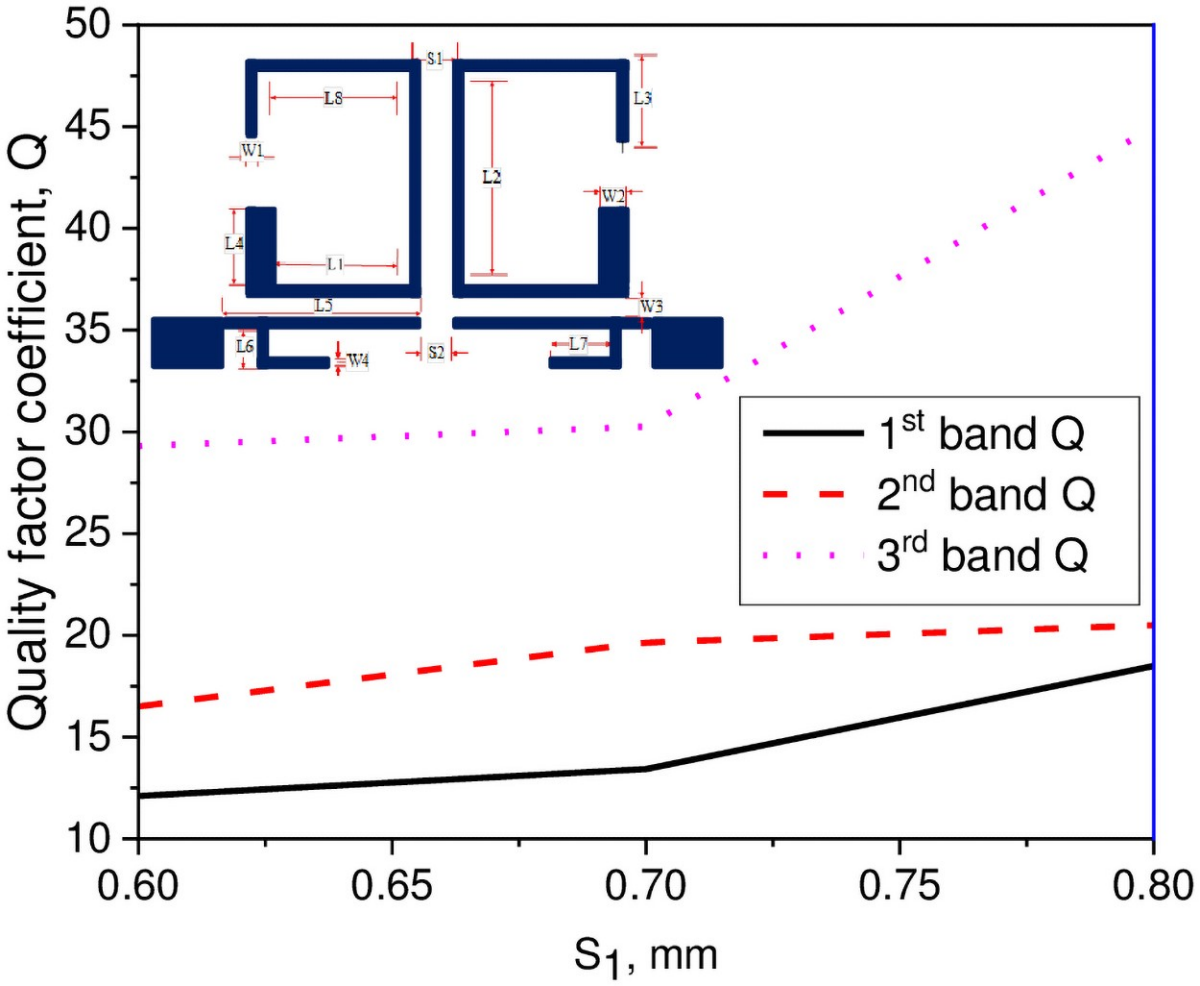
Graph of quality factor coefficient with S_1_ gap.

**Fig 6:**
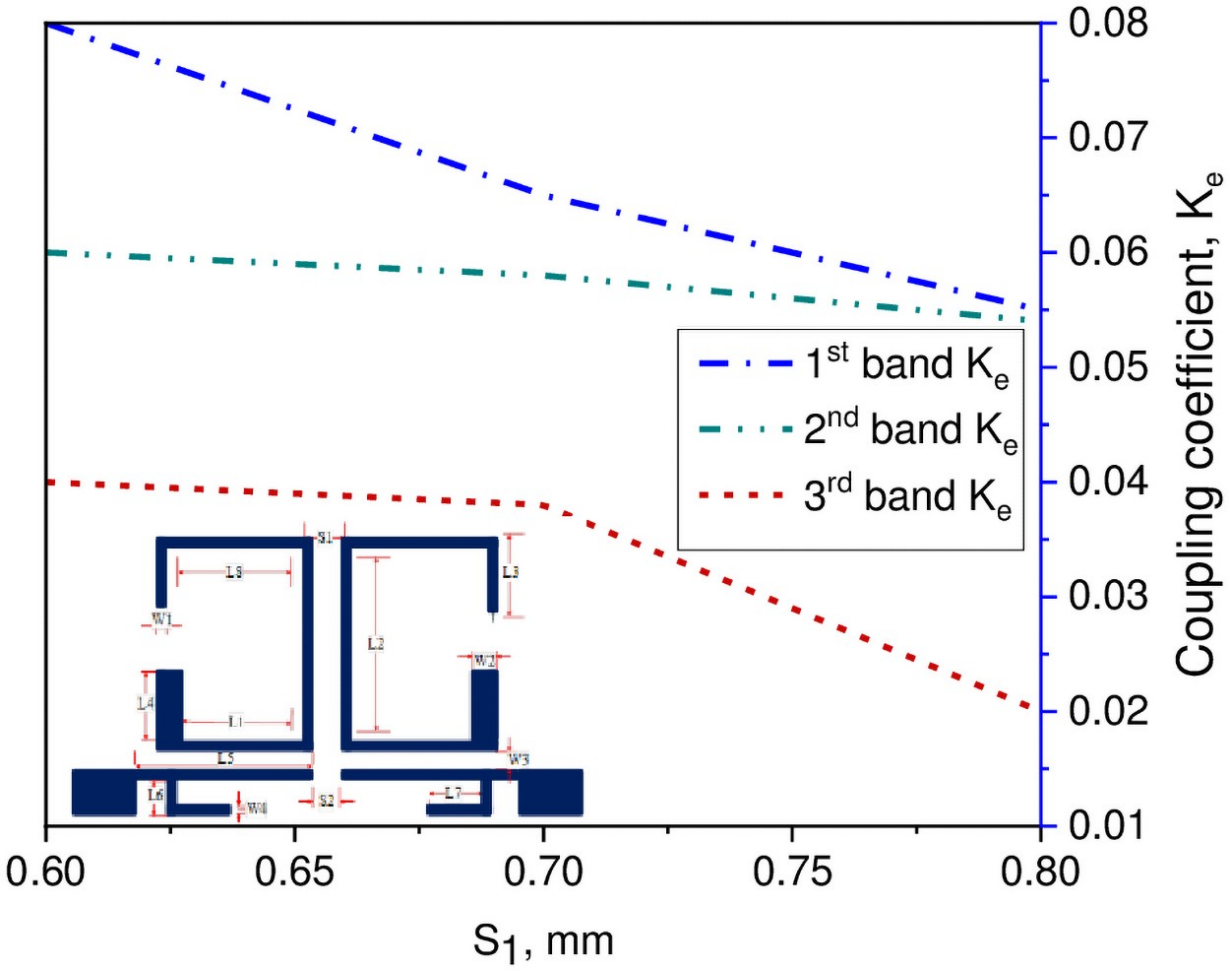
Graph of coupling coefficient with S_1_ gap.

**Fig 7:**
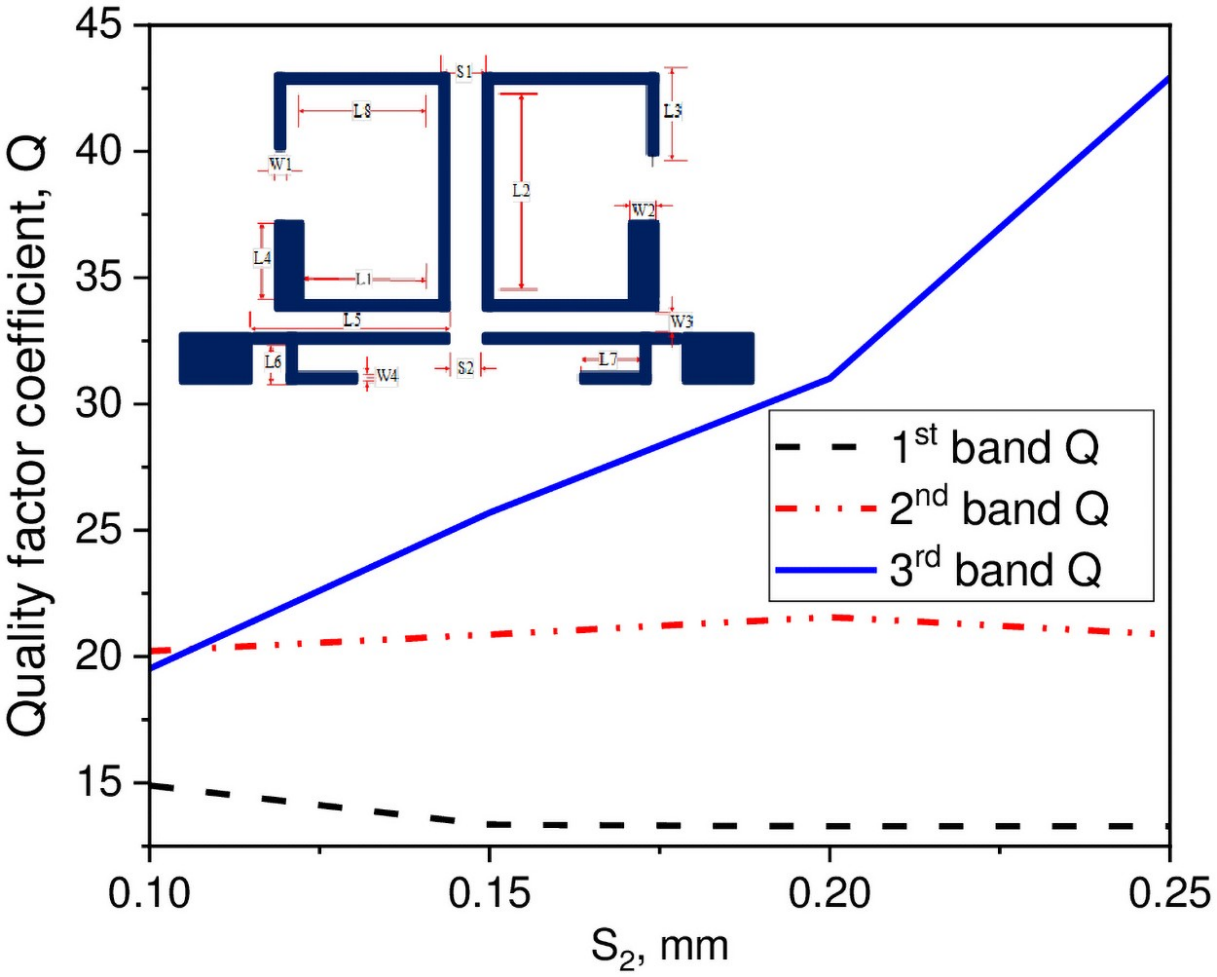
Graph of quality factor coefficient with S_2_ gap.

In above equations, f_l_ and f_h_ demonstrate the lower and upper resonance frequency mode of the asymmetric SIRs, f_c_ represents the resonant mode frequency, and FBW denotes the fractional bandwidth in percentage. Combining with the design specifications of the filter, herein, the Qi and K_i_ can be determined with respect to W_3_ gap as K_1_ = 0.075 and Q_1_ = 13.43 for the first passband, K_2_ = 0.027 and Q_2_ = 25 for the second passband, and K_3_ = 0.028 and Q_3_ = 46.26 for the third passband. Similarly, for S_1_ gap the K_1_ = 0.065 and Q_1_ = 13.43 for the first passband, K_2_ = 0.058 and Q_2_ = 19.65 for the second passband, and K_3_ = 0.038 and Q_3_ = 30.26 for the third passband. Tables 2 and 3 show the recorded EM simulated results of quality factor and coupling coefficient of each band for different values of W_3_, while the corresponding graphical plots are shown in Figs 3 and 4, respectively. Similarly, Tables 4 and 5 show the EM simulated results of quality factor and coupling coefficient of each band for different values of S_1_, while its graphical plots are shown in Figs 5 and 6, respectively. Moreover, the tap position of the feeding lines greatly affects the external quality factor of the third band. In Fig 7 and Table 6, it is clearly show that the external quality factor of the third band abruptly increases when the gap S_2_ varies from 0.1 mm to 0.25 mm, while the external quality factor of the first and second band remains unchanged. Moreover, the coupling between the TL and ASIR is calculated on the basis of standard design procedure given in [31], so, the coupling matrixes M_ij_ at 3.6 GHz and M_ij_ at 6.6 GHz of the proposed filter are derived as follows:

$${\text{M}}_{\text{ij}}=\begin{array}{cccc} 0 & 0.075 & 0.027 & 0\\ 0.075 & 0 & 0 & 0.027\\ 0.027 & 0 & 0 & 0.026\\ 0 & 0.027 & 0.028 & 0 \end{array}\;\text{for}\;3.6\;\text{GHz}$$ (14)

$${\text{M}}_{\text{ij}}=\begin{array}{cccc} 0 & 0.065 & 0.058 & 0\\ 0.065 & 0 & 0 & 0.058\\ 0.058 & 0 & 0 & 0.058\\ 0 & 0.058 & 0.038 & 0 \end{array}\;\text{for}\;6.6\;\text{GHz}$$ (15)

where M_ij_ is the coupling coefficient and

$${\text{M}}_{34}=\text{FBW}\times {\text{J}}_{2}/{\text{g}}_{2}$$ (16)

$${\text{M}}_{12}=\text{FBW}\times {\text{J}}_{1}/{\text{g}}_{1}$$ (17)

$${\text{M}}_{13}={\text{M}}_{24}=\text{FBW}/\surd {\text{g}}_{1}{\text{g}}_{2}$$ (18)

Where J_1_ = -0.276 and J_2_ = 0.932 are the admittance inverter constant and g_1_ = 1.276 and g_2_ = 1.3293 are the element values of the filter prototype.

**Table 2:** Simulated result of quality factor for different values of W_3_.

W_3_ (mm)	1^st^ band (GHz)	2^nd^ band (GHz)	3^rd^ band (GHz)
0.2	12.1	22.1	45
0.25	13.43	25	46.26
0.3	14.62	41.34	47.89
0.35	14.83	47.51	50.43

**Table 3:** Simulated result of coupling coefficient for different values of W_3_.

W_3_ (mm)	1^st^ band (GHz)	2^nd^ band (GHz)	3^rd^ band (GHz)
0.2	0.08	0.04	0.03
0.25	0.075	0.027	0.028
0.3	0.049	0.021	0.013
0.35	0.031	0.013	0.01

**Table 4:** Simulated result of quality factor for different values of S_1_.

S_1_ (mm)	1^st^ band (GHz)	2^nd^ band (GHz)	3^rd^ band (GHz)
0.6	12.1	16.5	29.3
0.7	13.43	19.65	30.26
0.8	18.5	20.5	45

**Table 5:** Simulated result of coupling coefficient for different values of S_1_.

S_1_ (mm)	1^st^ band (GHz)	2^nd^ band (GHz)	3^rd^ band (GHz)
0.6	0.08	0.06	0.04
0.7	0.065	0.058	0.038
0.8	0.055	0.054	0.02

**Table 6:** Simulated result of quality factor for different values of S_2_.

S_2_ (mm)	1^st^ band (GHz)	2^nd^ band (GHz)	3^rd^ band (GHz)
0.1	14.9	20.22	19.52
0.15	13.35	20.875	25.7
0.2	13.28	21.56	31.01
0.25	13.28	20.875	42.92

From the above discussion, it is verified that when the gap between the two resonators increases, the coupling coefficient decreases, and quality factor increases according to the Eqs 12 and 13.

## Results and discussion

**Fig 8:**
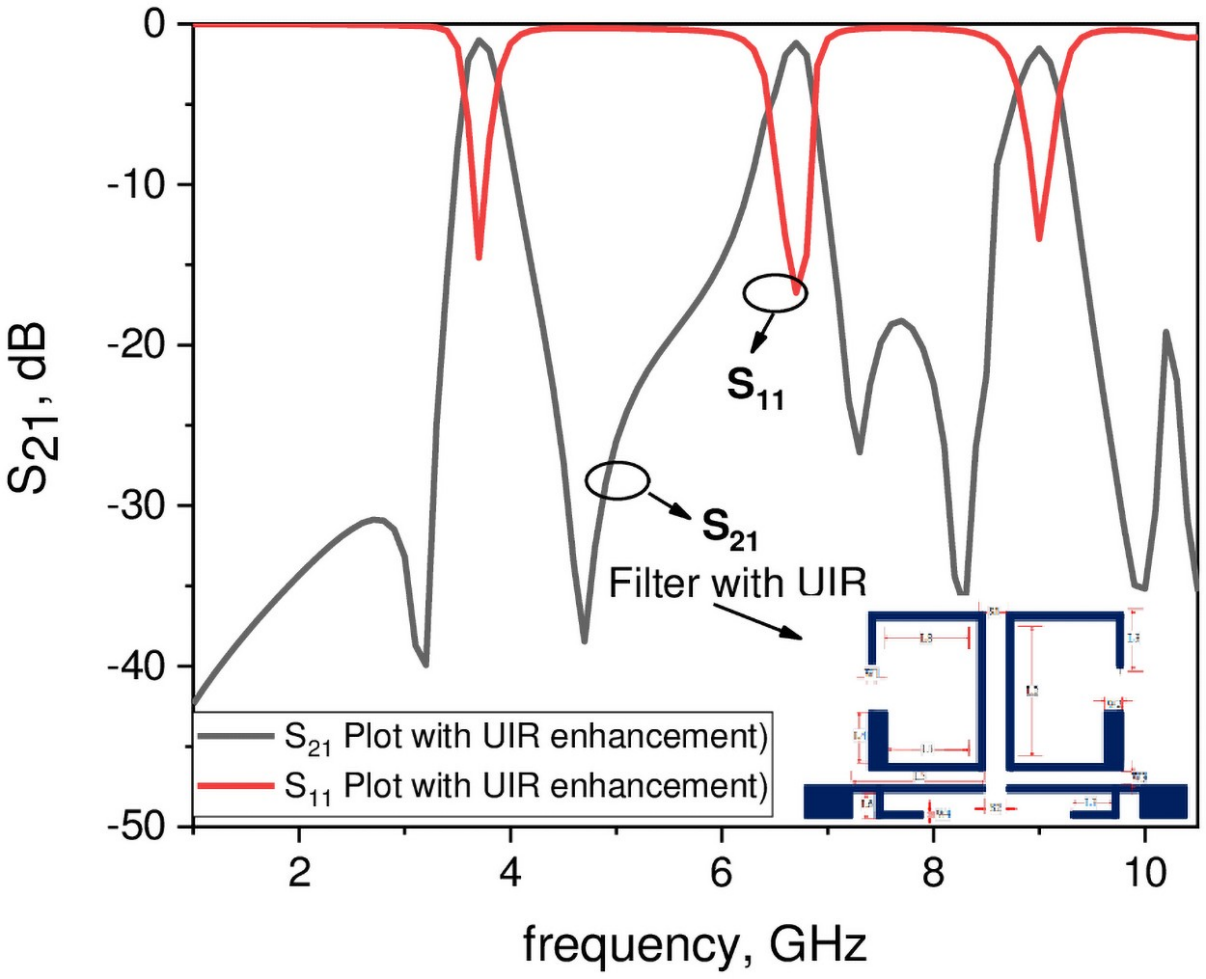
Frequency plots of the filter with uniform impedance resonator (UIR).

**Fig 9:**
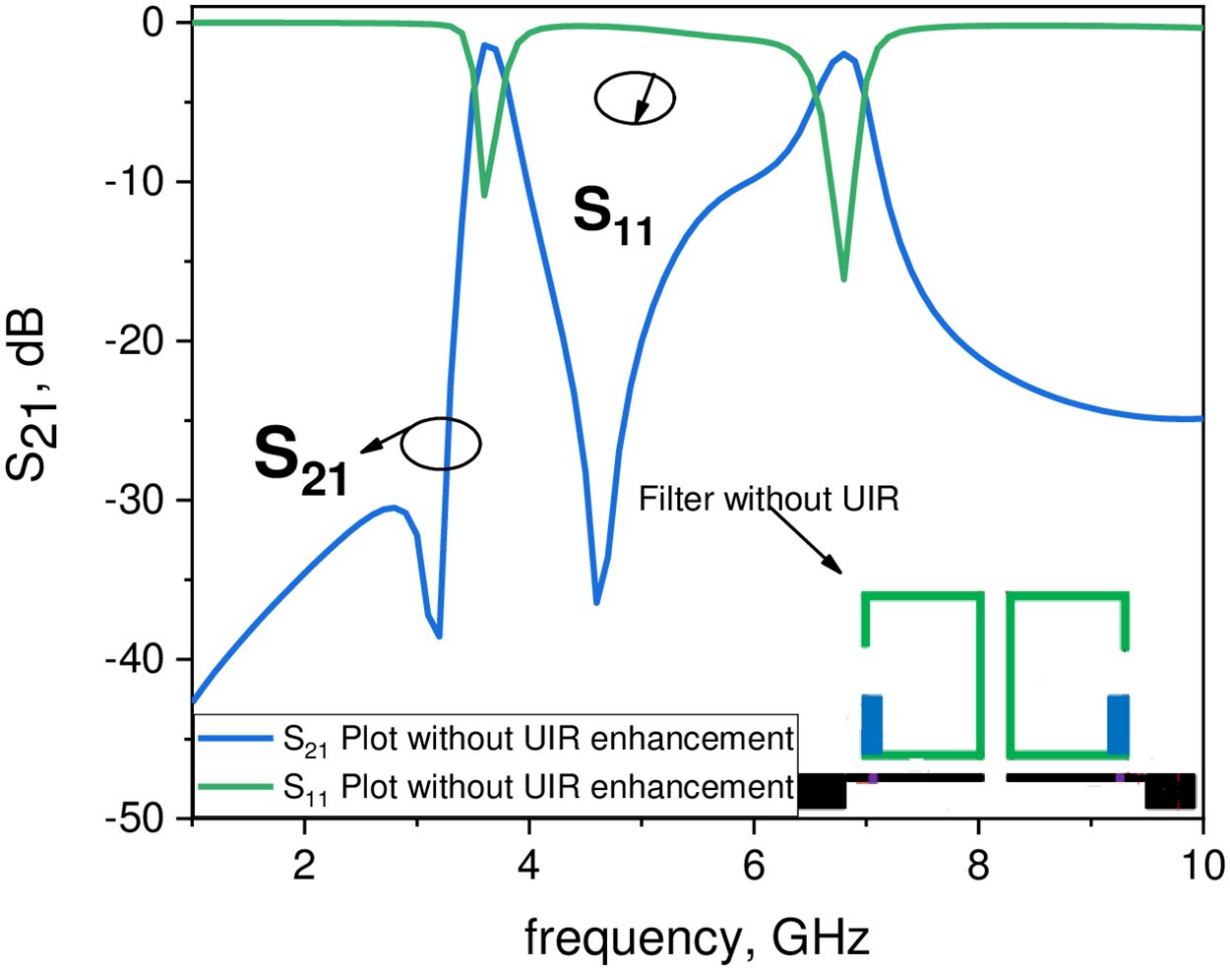
Frequency plots of the filter without uniform impedance resonator (UIR).

**Fig 10:**
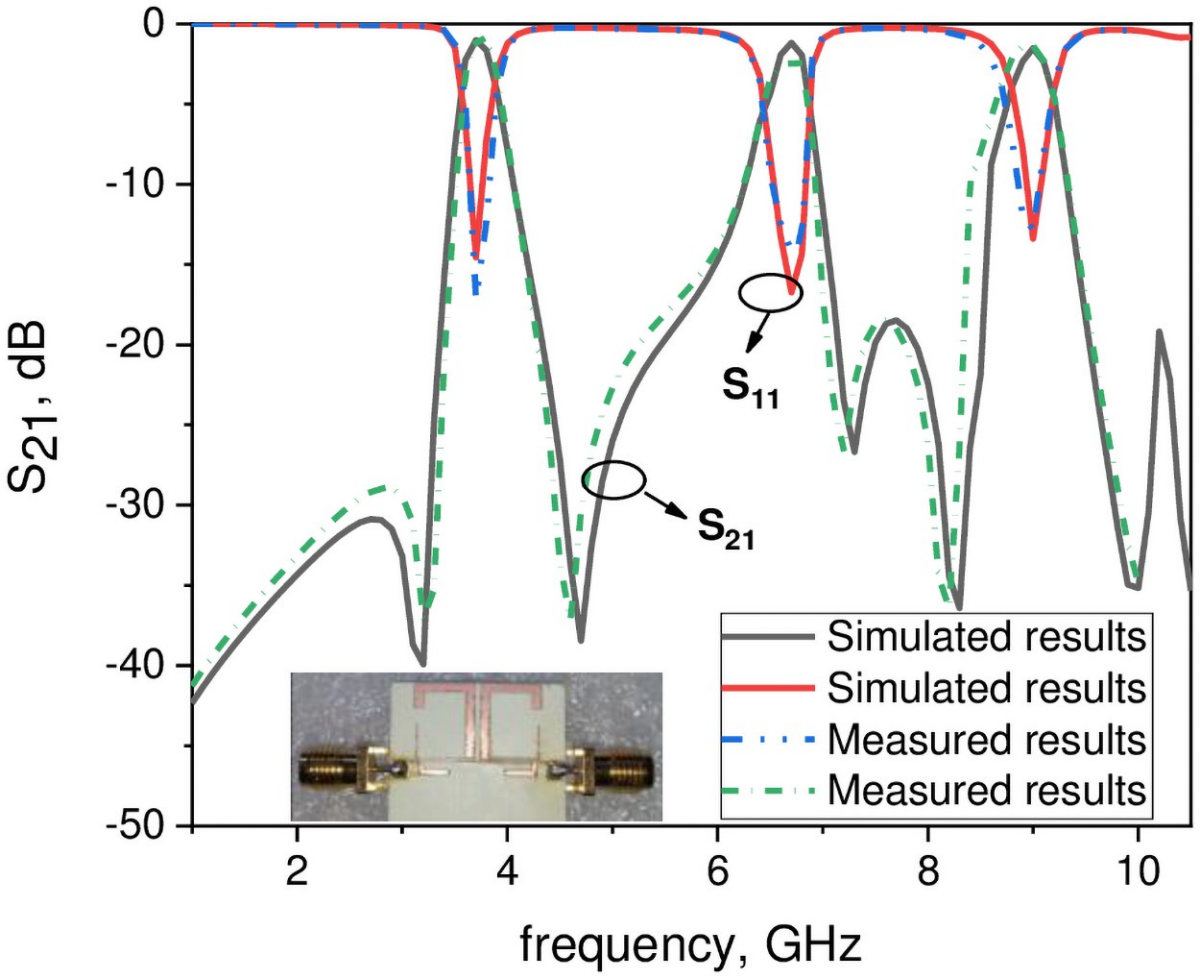
Simulated and measured resonance frequency plots of the proposed filter.

In this work the first two passbands are generated through the asymmetric coupled SIRs by choosing the design parameters consist of high impedance section (Z_1_ = 123.5 Ω, θ_1_ = 81.2°) with a strip width of 0.23 mm and low impedance section (Z_2_ = 55.8 Ω, θ_2_ = 195.3°) with a strip width of 1.4 mm, respectively. The resonant frequency ratio (f_2_/f_1_ = 1.83 and f_3_/f_1_ = 2.5) and the impedance ratio R = 0.45 with the physical length ratio α = 0.71 are selected for the proposed asymmetric resonator to generate the two passbands at frequency of 3.7 GHz and 6.6 GHz. The third passband is obtained by the uniform impedance resonator (UIR) centered at 9 GHz. Figs 8 and 9 show, the filter response with and without the UIR, which clearly reveal that the third passband is obtained by the UIR. Fig 10 demonstrate the simulated and measured frequency response along with a fabricated photograph of the proposed tri-band filter. This shows that the fabricated filter resonates at f_1_ = 3.7 GHz, f_2_ = 6.6 GHz, and f_3_ = 9 GHz for WiMAX and RFID wireless applications with the 3-dB fractional bandwidth (FBW_1_ = 7.52% for the first passband, FBW_2_ = 5.1% for the second passband, and FBW_3_ = 4.44% for the third passband), respectively. The minimum insertion loss (-20 log|S_21_|) is 0.99 dB for 3.7 GHz, 1.17 dB for 6.6 GHz, and 1.50 dB for 9 GHz, while the return loss (-20 log|S_11_|) is greater than 10 dB for the three passbands, respectively. The coupling between the two resonators denoted by S_1_ and the gap W_3_ between the pair of asymmetric SIRs and the input/output ports generates five transmission zeros at 3.19 GHz, 4.71 GHz, 7.72 GHz, 8.3 GHz, and 9.91 GHz between the passbands and thus high selectivity is obtained. Moreover, the space S_1_ should be minimum for achieving lower insertion loss. Furthermore, Table 7 summarizes the comparison of the proposed triple-band BPF with other state of the art filters in the literature, which proves that the presented filter has low insertion loss, wide bandwidth, compact size, and has a potential to be utilized in WiMAX, RFID and other triband wireless applications [18–29].

**Table 7:** Comparison between the proposed work with other latest triband filters.

Ref. No	CF (GHz)	IL (dB)	FBW (%)	Size (λ_g_×λ_g_)
[18]	2.09/3.52/5.46	1.18/0.54/0.88	11.3/20/12.1	0.12×0.42
[19]	0.83/1.57/1.88	0.9/1.7/0.8	NA	0.21×0.15
[20]	3.3/6/9	3.06/2.71/3.16	3/4.7/3.5	0.056
[21]	1.24/2.5/3.5	0.5/1.8/2.1	8/3.8/4.4	0.15×0.12
[22]	2.48/3.58/4.48	0.6/0.3/1.01	10/12.8/8	0.26×0.23
[23]	0.9/2.4/5.5	0.64/0.68/1.4	23/10/17	0.13×0.16
[24]	1.9/3.35/5.8	0.94/1.21/1.93	4.74/8.61/2.78	0.19×0.23
[25]	1.93/2.6/3.9	1.5/0.6/1.83	5/11/3	0.54×0.77
[26]	1.21/2.16/3.1	0.8/0.9/1.2	NA	0.14×0.20
[27]	2.4/3.5/5.15	1.6/1.6/1	6/5/7	0.23×0.19
[28]	2.4/3.5/5.2	1.2/1.8/1.5	6.3/4.4/5.9	0.049
[29]	1.8/2.4/3.5	1.2/1.8/2.1	7.8/3.7/2.9	0.23×0.20
**This work**	**3.7/6.6/9**	**0.99/1.17/1.50**	**7.52/5.1/4.44**	**0.02×0.03**

## Conclusions

In this article, a pair of asymmetric step impedance resonator with one-step discontinuity and open-circuited uniform impedance resonator is utilized to achieve a compact tri-band response centered at 3.7 GHz, 6.6 GHz, and 9 GHz for WiMAX and RFID wireless applications with the 3-dB FBW 3.52%, 5.1%, and 4.44%, respectively. The proposed filter has good in-band insertion loss for all three pass bands, i.e. 0.99 dB, 1.17 dB, and 1.50 dB, and the return loss is greater than 10 dB. By choosing the appropriate impedance ratio (R) and the physical length ratio (α) of the asymmetrical SIR, the filter can be perfectly tuned. The simulated results are in good agreement with the measurement results. Measurement results shows that the proposed three-band bandpass filter has high bandwidth, low insertion loss and compact size and can be widely used in modern high-performance multi-service wireless communication systems.

## Supporting information

S1 File (XLSX)
